# Glucose: an energy currency and structural precursor in articular cartilage and bone with emerging roles as an extracellular signaling molecule and metabolic regulator

**DOI:** 10.3389/fendo.2012.00153

**Published:** 2012-12-17

**Authors:** Ali Mobasheri

**Affiliations:** Faculty of Medicine and Health Sciences, School of Veterinary Medicine and Science, University of NottinghamNottingham, UK

**Keywords:** glucose, extracellular signaling, articular cartilage, bone, glucosensing, hexokinase, glucose transport, osteocalcin

## Abstract

In the skeletal system glucose serves as an essential source of energy for the development, growth, and maintenance of bone and articular cartilage. It is particularly needed for skeletal morphogenesis during embryonic growth and fetal development. Glucose is vital for osteogenesis and chondrogenesis, and is used as a precursor for the synthesis of glycosaminoglycans, glycoproteins, and glycolipids. Glucose sensors are present in tissues and organs that carry out bulk glucose fluxes (i.e., intestine, kidney, and liver). The beta cells of the pancreatic islets of Langerhans respond to changes in blood glucose concentration by varying the rate of insulin synthesis and secretion. Neuronal cells in the hypothalamus are also capable of sensing extracellular glucose. Glucosensing neurons use glucose as a signaling molecule to alter their action potential frequency in response to variations in ambient glucose levels. Skeletal muscle and adipose tissue can respond to changes in circulating glucose but much less is known about glucosensing in bone and cartilage. Recent research suggests that bone cells can influence (and be influenced by) systemic glucose metabolism. This focused review article discusses what we know about glucose transport and metabolism in bone and cartilage and highlights recent studies that have linked glucose metabolism, insulin signaling, and osteocalcin activity in bone. These new findings in bone cells raise important questions about nutrient sensing, uptake, storage and processing mechanisms and how they might contribute to overall energy homeostasis in health and disease. The role of glucose in modulating anabolic and catabolic gene expression in normal and osteoarthritic chondrocytes is also discussed. In summary, cartilage and bone cells are sensitive to extracellular glucose and adjust their gene expression and metabolism in response to varying extracellular glucose concentrations.

## INTRODUCTION

All living cells must be able to regulate their metabolic activity when faced with nutrient fluctuations in the extracellular environment (Mobasheri et al., 2008). Sensing the abundance and local fluctuations in the concentration of extracellular nutrients is a fundamental property of all living cells. Indeed, it has been suggested that it is an absolute requirement for the ability of living cells to adapt to changes in their environment (Shirazi-Beechey, 2005).

Nutrient-sensing is defined as a living cell’s ability to recognize and respond to fuel substrates and is essential for the survival of all prokaryotes and eukaryotes. Studies in plants (Rolland et al., 2002), yeast (Forsberg and Ljungdahl, 2001), and bacteria (Gilmore et al., 2003) have demonstrated that these organisms are able to sense and respond to changes in extracellular carbon and nitrogen metabolites. For example, in plants sugar-sensing allows photosynthesis to be switched off when carbohydrates are plentiful and turned on again when sugar levels are low (Rolland et al., 2002; Lejay et al., 2003). This adaptation involves hormonal regulation of gene expression and expressed protein function allowing the plant to make efficient and economic use of its energy stores.

In eukaryotic cells the physiological maintenance of nutrient and metabolite homeostasis is crucial to many fundamental cellular functions (Rolland et al., 2001). These functions include division, proliferation, differentiation, excitability, secretion, senescence (Nemoto et al., 2004), and apoptosis (Martens et al., 2005).

Each type of metabolic fuel used by living cells requires a distinct and carefully regulated uptake, storage, and utilization pathway involving transport, regulatory, and accessory molecules. In order to conserve valuable resources a cell will only produce the biomolecules that it requires at any one time. These requirements may change when cells engage in different activities such as division, proliferation, differentiation, and apoptosis. The quantity and type of nutrients and metabolic fuels that are available to a cell will also determine the complement of enzymes it needs to express from its genome for efficient utilization of the available nutrients. The uptake and storage of nutrients can also profoundly affect the size and morphology of cells.

Some metabolic fuels are also important structural precursors for the synthesis of other biochemicals and biological macromolecules. Glucose is an example of a metabolic fuel and a structural substrate for the synthesis of glycoproteins and glycoconjugates. Specific receptors on the cell membrane are activated in the presence of specific fuel molecules communicate to the cell nucleus by means of biochemical signaling cascades. This mechanism allows cells to maintain awareness of the available nutrients in their environment in order to adjust their metabolism to utilize the available substrate molecules most efficiently.

## GLUCOSE AS A SIGNALING MOLECULE IN YEAST AND MAMMALIAN CELLS

It is now well established that glucose is an extracellular signaling molecule in *Saccharomyces cerevisiae* (Santangelo, 2006). Yeast cells possess elaborate mechanisms for sensing the availability and levels of glucose and other sugars. Sugar-sensing allows them to adjust cellular metabolism to best utilize the available resources and respond appropriately during periods of nutrient stress (Thevelein and de Winde, 1999).

Extracellular glucose and other sugars can also function as signaling molecules in mammalian cells, exerting transcriptional control over many different genes – this has resulted in the establishment of a discipline known as “nutrigenomics” (van Ommen and Stierum, 2002; van Ommen, 2004; Corthesy-Theulaz et al., 2005; Grayson, 2010). Nutrigenomics is an exciting scientific discipline that explores how genes interact with nutrients and how nutrients influence gene expression. It involves the study of the effects of foods and food constituents on gene expression (van Ommen and Stierum, 2002). This field of study has emerged because of the realization that the health effects of food-derived substances start at the molecular level (van Ommen and Stierum, 2002; van Ommen, 2004). However, long before the advent of nutrigenomics, it was known that nutrients have the capacity to influence gene expression in microorganisms. Cells generally adapt to alterations in the extracellular concentrations of any given nutrient by regulating its transport rate across the plasma membrane (and subsequently its storage and metabolism). Such adaptation is essential for numerous subcellular functions and may involve transcriptional control of transporter genes and cell surface sensors.

Extracellular glucose and other monosaccharides also alter mRNA and protein stability. Accordingly, the cells, tissues, and organs of living organisms must possess sophisticated molecular mechanisms for sensing extracellular glucose. Invariably, when this control is lost, glucose homeostasis is compromised, which is often followed by metabolic disease.

This focused review article discusses the importance of glucose as a universal energy currency and the molecular mechanisms involved in glucose sensing in the pancreas and the gut before focusing on glucose transport and metabolism in bone and cartilage and highlighting areas for future research.

## HEXOKINASE: AN INTRACELLULAR GLUCOSE SENSOR

The hexokinase glucose sensor concept was introduced over four decades ago (Matschinsky and Ellerman, 1968). Hexokinase (also known as glucokinase) is an evolutionarily conserved intracellular glucose sensor (Moore et al., 2003; Olsson et al., 2003; Rolland and Sheen, 2005). It functions as a glucose-phosphorylating enzyme, which is the first enzyme in glycolysis. It is a key regulator of energy expenditure in most cells as it catalyzes the first step in the metabolism of glucose but has also been proposed to be involved in sugar sensing and signaling in yeast and in plants (Moore et al., 2003; Olsson et al., 2003; Rolland and Sheen, 2005). Hexokinase converts glucose into glucose-6-phosphate and controls glycolytic flux and glucose oxidation in pancreatic β cells thus acting as an intracellular glucose sensor (Matschinsky, 1996).

## GLUCOSE TRANSPORTERS: PLASMA MEMBRANE GLUCOSE SENSORS AND CARRIERS

**FIGURE 1:**
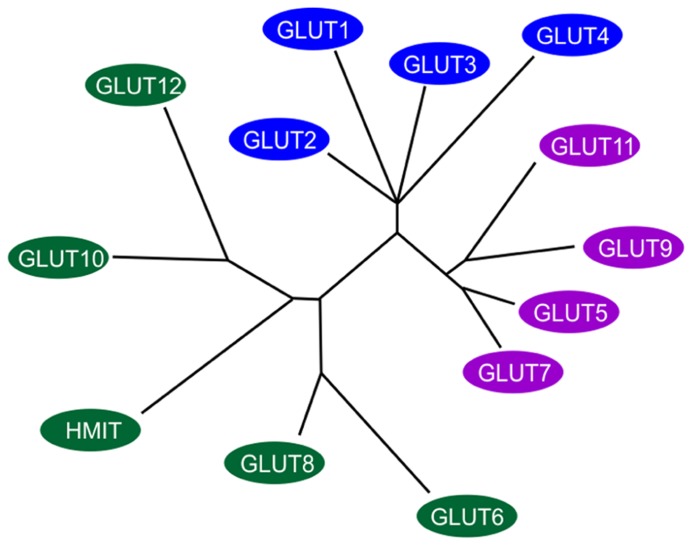
**Members of the extended GLUT/SLC2A family**. The radial phylogram was derived from a multiple sequence alignment of the 14 known members of the human GLUT/SLC2A family. The tree was constructed using neighbor-joining analysis of a distance matrix generated with PHYLIP software. The family is divided into three classes of GLUT proteins; class I includes GLUTs1-4 and GLUT14; class II includes the fructose transporter GLUT5, GLUT7, GLUT9 and GLUT11; class III includes GLUT6, GLUT8, GLUT10, GLUT12, and the H^+^-coupled myo-inositol transporter, HMIT. Adapted and modified from Wood and Trayhurn (2003).

The transport of sugar across the plasma membrane of mammalian cells is mediated by members of the GLUT/SLC2A family of facilitative sugar transporters and the SGLT/SLC5A family of Na^+^-dependent sugar transporters (Wood and Trayhurn, 2003). These proteins belong to a larger superfamily of proteins known as the major facilitator superfamily (MFS) or uniporter-symporter-antiporter family (Saier et al., 1999). The MFS family is one of the two largest families of membrane transporters in nature and accounts for nearly half of the solute transporters encoded within the genomes of microorganisms (bacteria, yeasts) and higher organisms such as plants and animals. The human genome project has identified fourteen members of the GLUT/SLC2A family, which have been cloned in humans (Wu and Freeze, 2002; Wood and Trayhurn, 2003; see **Figure 1**). Five of the mammalian facilitated glucose carriers (GLUTs 1–5) have been very well characterized but less is known about the remaining nine glucose carriers (GLUTs 6–14) since their discovery in late 2001 (Joost and Thorens, 2001) and much remains to be learned about their expression, tissue distribution, and transport functions (Uldry and Thorens, 2004).

## FUNCTIONAL ROLES OF THE GLUT PROTEINS

GLUT1, GLUT3, and GLUT4 are high-affinity transporters whereas GLUT2 is a low-affinity transporter; GLUT5 is primarily a fructose carrier (Thorens, 1996). High-affinity transporters are found in many metabolically active tissues, but their expression is higher in highly metabolic cells (i.e., hepatocytes, absorptive intestine epithelial cells, and proximal tubule cells; Tal et al., 1990; Thorens et al., 1990). GLUT1 is expressed in many human tissues including articular cartilage (Richardson et al., 2003; Mobasheri et al., 2005, 2008; Phillips et al., 2005; Peansukmanee et al., 2009; Rosa et al., 2009) and intervertebral disc (IVD; Richardson et al., 2008). IVD is anatomically and functionally very similar to cartilage although in contrast to cartilage it develops from notocordal cells rather than mesenchymal cells (Richardson et al., 2008). GLUT1 is abundantly expressed in the brain (Flier et al., 1987a), erythrocytes (Mueckler et al., 1985), and the liver, but is present in significantly lower quantities in cardiac and skeletal muscle which express other glucose transporters including GLUT3 (Guillet-Deniau et al., 1994; Hocquette and Abe, 2000; Shepherd et al., 1992) and GLUT4 (James et al., 1988; Charron et al., 1989). Elevated levels of the GLUT1 glucose transporter are induced by *ras* or *src* oncogenes (Flier et al., 1987b) and a role for this glucose transporter has been proposed in oncogenic transformation and tumor development ( Nagamatsu et al., 1993; Mellanen et al., 1994; Younes et al., 1996; Burstein et al., 1998; Semenza et al., 2001). GLUT2 is expressed in tissues carrying large glucose fluxes, such as the pancreas, intestine, kidney, and liver (Thorens, 1996), as well as in brain where it is involved in maintaining glucose homeostasis, and in cells where glucose-sensing is necessary (i.e., pancreatic β cells and hypothalamic neurons; Waeber et al., 1995). Indeed, in many experimental models of diabetes, GLUT2 gene expression is decreased in pancreatic β cells, which could lead to a loss of glucose-induced insulin secretion. As an adaptive response to variations in metabolic conditions, the expression of the GLUT1–5 transporters is regulated by glucose and different hormones (Thorens, 1996).

## GLUCOSE-SENSING VIA THE GLUT2 GLUCOSE TRANSPORTER AND HEXOKINASE

Early studies on glucose-sensing in the central nervous system suggested that the brain uses glucose-sensing units that are similar to the pancreas (Maekawa et al., 2000). Ependymal cells have been proposed to be putative glucose sensors in the brain. Immunohistochemical studies have shown strong hexokinase-like immunoreactivities in the ependymal cells, endothelial cells, and many serotonergic neurons (Maekawa et al., 2000). Ependymal cells were found to exhibit GLUT2 and GLUT1-like immunoreactivities on the cilia in addition to GLUT4-like immunoreactivity densely in the cytoplasmic area. These results raised the possibility that these cells form part of a more sophisticated array of glucose sensors in the brain.

The study of certain neuroendocrine cells outside the pancreatic islet has led to the suggestion that the intracellular glucose sensor hexokinase may play a broader role in intracellular glucose-sensing by neuroendocrine cells than was thought previously (Jetton et al., 1994) and more recent work has shown that electrogenic sugar entry via SGLT1 and SGLT3 provide a novel mechanism for glucose-sensing by neuroendocrine cells (Gribble et al., 2003).

## GLUCOSENSING AND TRANSPORT IN PANCREATIC β CELLS, ENTEROCYTES AND NEURONS

**FIGURE 2:**
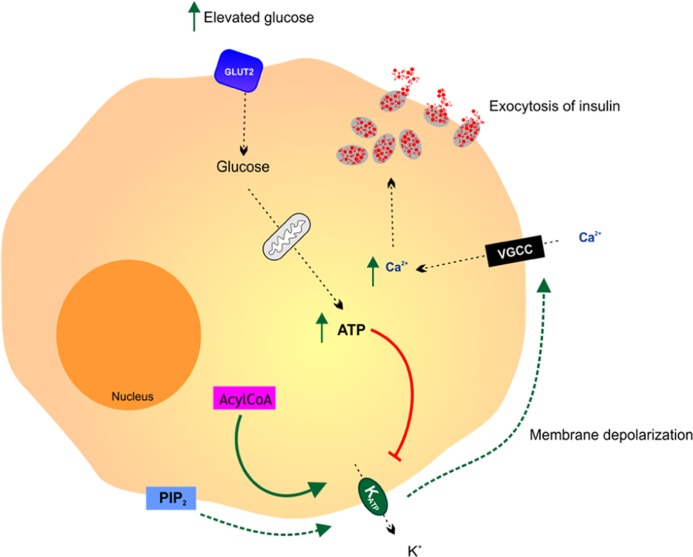
**Glucosensing in the pancreatic β cell**. A rise in blood glucose is an important metabolic signal that closes K_ATP_ channels, causing membrane depolarization, activation of voltage gated calcium channels (VGCC), free calcium entry and insulin release by exocytosis. It is thought that various additional effectors including phosphatidylinositol-4,5-bisphosphate (PIP2) and acyl CoAs modulate the ATP sensitivity of the K_ATP_ channel thereby affecting the coupling of pancreatic cell metabolism to insulin secretion. Adapted from Koster et al. (2005).

The pancreatic β cell is the archetypal glucose sensor (**Figure 2**; Efrat et al., 1994). The production and secretion of insulin by β cells in response to an increase in the level of blood glucose above 5 mM is central to the process of blood glucose homeostasis. The metabolism of glucose is essential for the process of glucose-sensing. The high-Km glucose transporter GLUT2 and the high-Km glucose phosphorylating enzyme hexokinase have been implicated in coupling insulin secretion to extracellular glucose levels (Efrat et al., 1994). Glucosensing in pancreatic β cell is one of the best models for the study of glucosensing and glucose-regulated processes in other tissues such as the gut, kidney, and the hypothalamus. The function of β cells is controlled by a glucose sensor that operates at physiological glucose concentrations and acts in synergy with signals that integrate messages originating from hypothalamic neurons and endocrine cells in the gut and the pancreas. Glucosensing neurons are specialized cells that use glucose as a signaling molecule to alter their action potential frequency in response to variations in ambient glucose levels.

**FIGURE 3:**
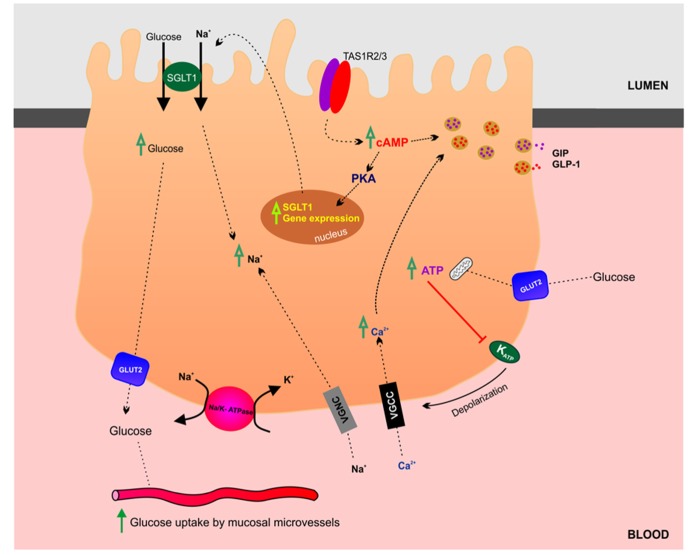
**Glucosensing and transport in the gut**. This schematic highlights the transport and regulatory mechanisms involved in glucose absorption by enterocytes in the small intestine.

In the gut luminal glucose is sensed by specialized chemosensitive cells scattered throughout the intestinal epithelium (Tolhurst et al., 2012). Enteroendocrine and tuft cells make direct contact with the gut lumen and release a range of chemical mediators, which can either act in a paracrine fashion interacting with neighboring cells and nerve endings or as classical circulating hormones. At the molecular level, the chemosensory machinery involves multiple and complex signaling pathways including activation of G-protein coupled receptors and solute carrier transporters (summarized in **Figure 3**). The sweet taste receptor (TAS1R3) and gustducin in the gut sense sugars by regulating the expression of SGLT1 (Margolskee et al., 2007). Sweet taste receptors in the small intestine have also been shown to stimulate glucose absorption through stimulation of apical GLUT2 in enterocytes (Mace et al., 2007). In summary, sugar-sensing by enterocytes combines membrane bound detectors and sugar metabolism (Le Gall et al., 2007). A detailed discussion of glucose-sensing in the gut and the biology of gustducin and taste receptors is beyond the scope of this review and readers are encouraged to refer to the relevant literature (Fujita et al., 2009; Reed and Margolskee, 2010).

Evidence exists that the extrapancreatic cells producing and secreting these (neuro)endocrine signals also exhibit a glucose sensor and an ability to integrate nutrient and (neuro)hormonal messages. Similarities in these cellular and molecular pathways provide a basis for a network of coordinated functions between distant cell groups, which is necessary for an appropriate control of nutrient homeostasis. The glucose sensor seems to be a fundamental component of these control mechanisms. Its molecular characterization is most advanced in pancreatic β cells, with important roles for glucokinase and mitochondrial oxidative fluxes in the regulation of ATP-sensitive K^+^ channels. Other glucose-sensitive cells in the endocrine pancreas, hypothalamus, and gut were found to share some of these molecular characteristics. It has been proposed that similar metabolic signaling pathways influence the function of pancreatic α cells, hypothalamic neurons, and gastrointestinal endocrine and neural cells.

Glucosensing neurons are specialized cells that use glucose as a signaling molecule to alter their action potential frequency in response to variations in ambient glucose levels. Hexokinase appears to be the primary regulator of most neuronal glucosensing, but other regulators almost certainly exist. Glucose-excited neurons increase their activity when glucose levels rise, and most use hexokinase and an ATP-sensitive K^+^ channel as the ultimate effector of glucose-induced signaling. Glucose-inhibited (GI) neurons increase their activity at low glucose levels. Although many use hexokinase, it is unclear what the final pathway of GI neuronal glucosensing is. Glucosensing neurons are located in brain sites and respond to and integrate a variety of hormonal, metabolic, neurotransmitter, and peptide signals involved in the regulation of energy homeostasis and other biological functions. Although it is still uncertain whether daily fluctuations in blood glucose play a specific regulatory role in these physiological functions, it is clear that large decreases in glucose availability stimulate food intake and counter-regulatory responses that restore glucose levels to sustain cerebral function.

It is also suggested that sweet taste signaling functions as a hypothalamic glucose sensor (Ren et al., 2009). The heterodimeric G-protein coupled sweet receptor TAS1R2/TAS1R3 has been proposed as a candidate membrane-bound brain glucosensor (Ren et al., 2009).

Finally, glucosensing is altered in obesity and after recurrent bouts of hypoglycemia, and this altered sensing may contribute to the adverse outcomes of these conditions. Thus, although a great deal is already known, much more remains to be learned about the physiological function of brain glucosensing neurons. It is possible that similar glucosensing mechanisms may operate in other organ systems.

## GLUCOSENSING AND TRANSPORT IN ARTICULAR CARTILAGE

Articular cartilage is a mechanically unique and resilient connective tissue responsible for load-bearing and low-friction movement in the synovial joints of all vertebrates (Buckwalter et al., 2005). The transport of nutrients (i.e., glucose, other hexose and pentose sugars, amino acids, nucleotides, nucleosides, and water-soluble vitamins such as vitamin C) into articular chondrocytes is essential for the synthesis of collagens, proteoglycans, and glycosaminoglycans (Clark et al., 2002; Mobasheri et al., 2002a; Goggs et al., 2005; McNulty et al., 2005). There are numerous biological mechanisms by which nutritional factors might be expected to exert favorable influences on cartilage function and pathophysiological events in disease processes including osteoarthritis (OA; McAlindon, 2006). A decade ago, very little was known about nutrient transport in chondrocytes, particularly the transport of glucose, related sugars and water-soluble vitamins, which are essential for the synthesis of glycosaminoglycans by chondrocytes. Ten years later we have gained some information about how glucose is transported into chondrocytes, but very little knowledge about how these cells sense extracellular glucose. However, we are beginning to understand more about the sensitivity of chondrocytes to extracellular glucose and how they adjust their gene expression and metabolism in response to varying extracellular glucose concentrations (Rosa et al., 2011).

Glucose is a crucial nutrient for cartilage function *in vivo* as it is for many other tissues and organs. However, it has always been assumed that glucose is important for the *in vitro* cultivation of chondrocytes, *ex vivo* maintenance of cartilage explants, and cartilage tissue engineering procedures. No one had actually studied the molecular mechanisms responsible for glucose and glucose-derived vitamins such as vitamin C until the early 1990s when Bird et al. (1990) and Hernvann et al. (1992), 1996 studied the kinetics of glucose transport by chondrocytes and synovial fibroblasts in the presence and absence of proinflammatory cytokines. By the late 1970s it was well established that ascorbic acid supplementation was essential for maintaining sulfated proteoglycan metabolism in chondrocyte cultures and growth plate metabolism, hypertrophy, and extracellular matrix mineralization (Schwartz and Adamy, 1977; Schwartz et al., 1981; Gerstenfeld and Landis, 1991). Studies by Otte and Hernvann related the importance of glucose as a metabolic substrate (Otte, 1991) and emphasized the fact that glucose uptake is stimulated by catabolic cytokines in chondrocytes (Hernvann et al., 1992) and that stimulated glucose uptake is inhibited by anti-inflammatory cortisol (Hernvann et al., 1992, 1996).

The expanded nomenclature of the GLUT/SLC2A family of glucose/polyol transporters in 2001/2002 (Joost and Thorens, 2001; Joost et al., 2002) stimulated our laboratory to investigate the expression of its newly identified members in chondrocytes. Published data from our laboratory went on to suggest that chondrocytes express multiple isoforms of the GLUT/SLC2A family (Mobasheri et al., 2002b,c, 2008; Richardson et al., 2003). In other tissues GLUT proteins are expressed in a cell-specific manner, exhibit distinct kinetic properties, and are developmentally regulated. Several GLUTs expressed in chondrocytes are regulated by hypoxia (Peansukmanee et al., 2009), hypoxia mimetics (Mobasheri et al., 2006a), insulin-like growth factors such as insulin-like growth factor I (IGF-I; Richardson et al., 2003; Phillips et al., 2005), and proinflammatory cytokines ( Phillips et al., 2005; Mobasheri et al., 2008).

The members of the extended GLUT family exhibit a surprisingly diverse substrate specificity (Joost and Thorens, 2001). However, despite the potential role of GLUT proteins in the uptake of glucose, fructose, ascorbate, and glucosamine, this aspect has not been investigated in chondrocytes and should be the focus of future research.

The interest in glucose transport and metabolism has now extended beyond the expression of GLUT/SLC2A family members and their regulation by hypoxia and inflammatory stimuli. There is now significant interest in the role of glucose as a signaling molecule and metabolic regulator in chondrocytes in health and disease. Chondrocytes are capable of adjusting to high and low glucose concentrations by changing the protein levels of GLUT1 (and perhaps other GLUT proteins as well; Rosa et al., 2009). This is a relatively simple concept that was put forward by the author in the first section of this review and in previous review articles published in 2002 (Mobasheri et al., 2002c) and 2006 (Mobasheri et al., 2006b). The rationale for this idea comes from the links between endocrinology, cartilage biology, and rheumatology. Endocrinological disorders such as diabetes mellitus are common among arthritis patients and vice versa (Rosenbloom and Silverstein, 1996). In diabetes, advanced glycation end products are thought to form as a result of non-enzymatic reaction of excess glucose with proteins in the extracellular matrix of a variety of connective tissues, including articular cartilage, causing stiffening and loss of biomechanical function (Rosenbloom and Silverstein, 1996). This clinically important fact has led to the idea that diabetes may actually favor the development and/or progression of OA. Rosa et al. (2009) hypothesized that chondrocytes may be able to sense and adjust to variations in the extracellular glucose concentration, resulting from hypoglycemia and hyperglycemia. The used high-density monolayer cultures of chondrocytes, isolated from normal and OA human cartilage, to compare the ability of normal and OA chondrocytes to regulate their glucose transport capacity in conditions of insufficient or excessive extracellular glucose. This work was done to identify the putative mechanisms involved and the eventual deleterious consequences of excessive glucose, namely the production of reactive oxygen species (ROS). They found that normal human chondrocytes are able to adjust to variations in the extracellular glucose concentration by modulating GLUT1 synthesis and degradation. However, OA chondrocytes exposed to high glucose were unable to down-regulate GLUT1. OA-derived chondrocytes accumulated more glucose and produced more ROS. The authors concluded that impaired GLUT1 down-regulation might constitute an important pathogenic mechanism by which diabetic conditions characterized by hyperglycemia can promote degenerative changes, thus facilitating the progression of OA.

More recent *in vitro* work by the same group has demonstrated that extracellular glucose can modulate anabolic and catabolic gene expression in normal and osteoarthritic human chondrocytes (Rosa et al., 2011). The authors used real time RT-PCR to demonstrate that extracellular glucose concentration can modulate the expression of genes encoding collagen type I, collagen type II, and matrix metalloproteinases MMP-1 and MMP-13. Exposure to high glucose (30 mM) increased the mRNA levels of both MMP-1 and MMP-13 in OA-derived chondrocytes, whereas in normal chondrocytes only MMP-1 increased. Incubating chondrocyte cultures with transforming growth factor-β (TGF-β), a pro-anabolic and chondrogenic growth factor, down-regulated MMP-13 gene expression. However, exposure to high glucose for 24 h blocked the TGF-β-induced down-regulation of MMP-13 gene expression, while the inhibitory effect of TGF-β on MMP-1 expression was only partially reduced. Therefore, exposure of human chondrocytes to high glucose appears to favor catabolic gene expression and degradative signaling pathways in chondrocytes.

These recent studies highlight the fact that chondrocytes sense and respond to changes in the concentration of extracellular glucose. There are also age-related changes in this sensing and adaptation phenomenon as the ability of chondrocytes to adjust to high glucose concentrations is lost in aging/OA chondrocytes. The fact that this ability is compromised in aged/OA chondrocytes is an important finding and suggests that these metabolic alterations favor oxidative stress and catabolic gene expression.

## GLUCOSENSING AND TRANSPORT IN BONE

Evidence for GLUT expression and glucose transport in bone and bone-derived cells is relatively scant. A recent PubMed with the keywords “glucose transporter, bone and GLUT” yielded a limited number of articles. The earliest article was published in 1996. In this paper by Thomas et al. (1996), GLUT1 expression (mRNA and protein) was demonstrated in UMR 106-01 (a clonal rat osteosarcoma cell line that displays many osteogenic and osteoblastic features) by studying the effects of dexamethasone on glucose metabolism. There is evidence demonstrating the expression of GLUT1 and GLUT4 in murine models of endochondral bone formation as well as their role in the bone formation process (Maor and Karnieli, 1999). GLUT 1–5 expression has been demonstrated independently by another group in a murine model of endochondral bone formation (Ohara et al., 2001). There is also evidence for GLUT1 isoform expression in human osteosarcoma cell lines (Fan et al., 2010; Cifuentes et al., 2011) and rodent osteoblastic (PyMS) cells in which glucose transport is regulated by parathyroid hormone and insulin-like growth factor 1 (IGF-1; Zoidis et al., 2011). It is becoming increasingly apparent that glucose transport and metabolism is important for bone cell function and bone itself has the capacity to influence systemic glucose metabolism. The following sections discuss these emerging concepts in greater detail.

## THE RELEVANCE OF GLUCOSE METABOLISM IN BONE REMODELING

Bone remodeling is a continuous process that involves old bone resorption and new bone formation. Bone remodeling occurs normally, as a physiologically regulated process during growth, development, and adaptation to mechanical load and physical exercise. Bone remodeling controls the reshaping and replacement of bone following injuries such as fractures but also following micro-damage, which is known to occur during intensive physical activity. Remodeling effectively responds to the functional demands of mechanical loading, in coordination with endocrine signals. An imbalance in the regulation of bone resorption and bone formation results in metabolic bone diseases such as osteoporosis (Rosen, 2000). It can also occur as a consequence of chronic joint disease; for example subchondral sclerosis is associated with age-related joint degeneration (Burr and Gallant, 2012). Bone remodeling is accomplished by the metabolically active osteoblasts and osteoclasts. Osteoblasts are osteogenic cells of mesenchymal origin and osteoclasts are giant multinucleated bone-resorbing cells that arise from the fusion of monocytes/macrophages (Teitelbaum, 2000; Del Fattore et al., 2012). Recent studies have highlighted that glucose metabolism is important for bone remodeling. The following section discusses the emerging role of osteocalcin in osteoblasts mediated glucose homeostasis.

## THE ROLE OF BONE-SECRETED OSTEOCALCIN IN GLUCOSE HOMEOSTASIS

**FIGURE 4:**
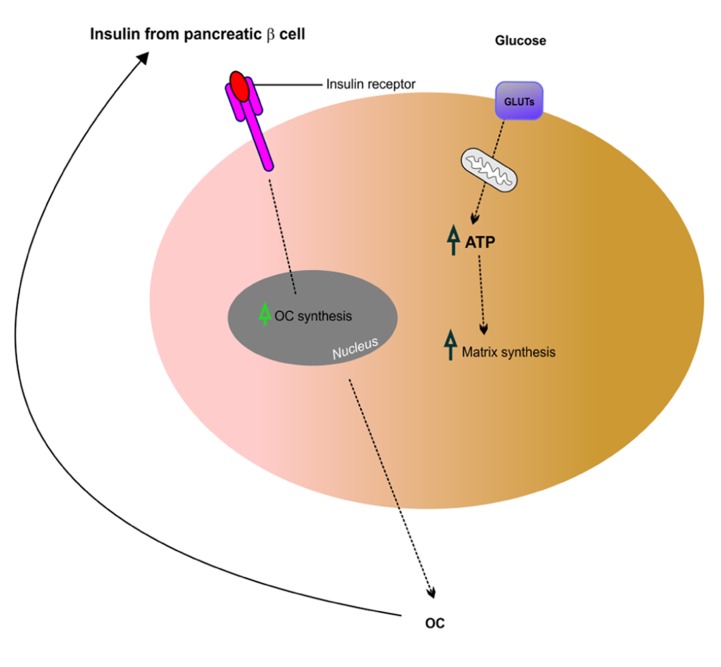
**Putative mechanism of insulin signaling and osteocalcin regulation in osteoblasts**. Insulin released from pancreatic β cells binds the insulin receptor (InsR) on osteoblasts, which increases osteocalcin (OC) synthesis. This feeds back in a forward loop to β cells thus stimulating insulin secretion and also regulating glucose homeostasis potentially via GLUTs expressed in osteoblasts. Adapted from Fulzele and Clemens (2012).

Recent work suggests that insulin signaling in osteoblasts integrates remodeling and energy metabolism in bone (Ferron et al., 2010; Rosen and Motyl, 2010). Glucose metabolism in osteoblasts is regulated through the action of osteocalcin, a hormone that becomes activated by uncarboxylation. Elegant studies by Ferron et al. (2010) have shown that insulin signaling in osteoblasts is necessary for whole-body glucose homeostasis through up-regulation of osteocalcin expression and activity. Similar work carried out by Fulzele et al. (2010) has shown that insulin receptor signaling in osteoblasts regulates postnatal bone acquisition and body composition. These two studies support the hypothesis that bone remodeling is intimately linked to glucose metabolism and homeostasis. Insulin signaling and action on bone-forming osteoblasts promotes their activation and enhances the production of osteocalcin, a secreted mediator of insulin sensitivity, through modulation of bone resorption. These studies also highlight existence of a bone–pancreas endocrine loop through which insulin signaling in the osteoblast ensures osteoblastic differentiation and stimulates osteocalcin production, which in turn regulates insulin sensitivity and pancreatic insulin secretion (see **Figure 4**). By expressing insulin receptors osteoblasts bind insulin and control their own development. Hence, insulin signals in osteoblasts activate osteocalcin and promote glucose metabolism. In summary, osteocalcin is a new regulator of glucose metabolism in bone through increasing insulin secretion from the pancreas and enhancing insulin sensitivity in peripheral tissues. This recent finding reinforces the fact that bone is intimately involved in regulating glucose and lipid metabolism. Therefore, complex interactions between bone and the pancreas integrate bone remodeling and glucose metabolism (Ng, 2011). Insulin and osteocalcin are key molecular links between bone remodeling and energy metabolism. The osteoblast is an important cellular target of insulin action and is used to control whole-body glucose homeostasis and energy expenditure and bone resorption is the mechanism regulating osteocalcin activation (Clemens and Karsenty, 2011). Clearly, this is a rapidly expanding field and recent studies that are beyond the scope of this review suggest that in addition to osteocalcin, other osteoblast-derived hormones such as adiponectin (Bacchetta et al., 2009) may contribute to the emerging function of the skeleton as a regulator of energy metabolism (Yoshikawa et al., 2011).

## THE EFFECT OF GLUCOSE ON OSTEOBLASTOGENESIS AND OSTEOCLASTOGENESIS

Osteoblastogenesis and osteoclastogenesis refer to the formation of osteoblasts and osteoclasts respectively. These two processes may be regulated by glucose itself, at the metabolic substrate level. Hyperglycemia has been implicated in the pathogenesis of diabetic bone disease. To examine the effects of glucose on osteoclastogenesis, Wittrant et al. (2008) studied the effect of high d(+)glucose on RANKL-induced osteoclastogenesis using RAW264.7 cells and bone marrow macrophages (BMM) as *in vitro* models of bone resorption. The authors showed that high d(+)glucose concentrations inhibit RANKL-induced osteoclastogenesis. High d(+)glucose inhibits osteoclast formation, ROS production, caspase-3 activity, and migration in response to RANKL through a metabolic pathway. Their findings also suggest that high d(+)glucose may alter RANKL-induced osteoclast formation by inhibiting redox-sensitive NF-kappaB activity through an anti-oxidative mechanism. This study increases our understanding of the role of glucose in diabetes-associated bone disease. This data suggest that high glucose levels may alter bone turnover by decreasing osteoclast differentiation and function in diabetes and provide new insight into the biologic effects of glucose on osteoclastogenesis.

Mature osteoclasts rely on the citric acid cycle and mitochondrial respiration to generate high levels of ATP production for acid secretion and bone resorption. A study by Kim et al. (2007), has reported that glycolysis, oxidative phosphorylation, and lactate production are increased during receptor activator of nuclear factor-kappaB ligand (RANKL)-induced osteoclastogenesis in RAW264.7 and bone marrow-derived macrophage cells. This study indicates that glucose metabolism is increased during osteoclast differentiation resulting in a metabolic shift toward accelerated glucose metabolism at an early stage of RANKL-stimulated osteoclast differentiation. Increased mitochondrial oxidative phosphorylation will then result in elevated ATP production and enhanced osteoclast differentiation. Taken together, these studies indicate that there is a link between hyperglycemia and osteoclastogenesis.

The link between diabetes mellitus and the loss of bone mineral density and quality has focused research on the effects of glucose on osteoclastogenesis. Consequently, there has been less focus on the regulation of osteoblastogenesis by glucose. Zhen et al. (2010) studied the effects of the oral anti-diabetic drug metformin on rat primary osteoblasts and found that this drug reverses the deleterious effects of high glucose on osteoblast function. High concentrations of glucose reduced cellular proliferation, alkaline phosphatase activity and calcium deposition. In contrast high glucose significantly increased ROS and apoptosis in a dose-dependent manner. Metformin reversed the effects of high glucose by increasing cellular proliferation, alkaline phosphatase activity, and calcium deposition, as well as inhibiting ROS and apoptosis. These findings suggest that glucose is a regulator of osteoblastogenesis since it affects osteoblast formation and function in a dose-dependent manner.

Osteoblastogenesis and osteoclastogenesis contribute to bone remodeling and since both cell types are under the control of multiple hormones, there are also exciting opportunities for studying how the processes of bone turnover and remodeling are regulated by metabolic hormones such as insulin and leptin. For example, bone formation by osteoblasts is negatively regulated by leptin, which is secreted by adipocytes. Leptin deficiency leads to increased osteoblast activity and increased bone mass. In contrast, osteocalcin acts as a regulator of insulin in the pancreas and adiponectin in the adipocyte to modulate energy metabolism. Osteocalcin deficiency in knockout mice leads to decreased insulin and adiponectin secretion, insulin resistance, higher serum glucose levels, and increased adiposity (Wolf, 2008). These links highlight the importance of glucose metabolism and insulin action in bone and how insulin signaling in osteoblasts contributes to whole-body glucose homeostasis by increasing the expression and activity of osteocalcin.

## CONCLUDING REMARKS AND FUTURE RESEARCH PRIORITIES

The integrity of bone and articular cartilage is maintained via the finely tuned interaction between the cells in these tissues and systemic, paracrine and endocrine mediators. Disruption of homeostatic processes involved in bone and cartilage turnover can lead to a variety of diseases including osteoporosis and osteoarthritis. Although various regulatory and signaling systems are known to be involved in maintaining bone and cartilage health, our basic understanding of these processes is limited compared to other tissues and organs. This review has highlighted that glucose is a source of energy and a structural precursor for extracellular matrix production in bone and cartilage. Glucose is also an important but neglected signaling molecule in articular cartilage and bone. Recent studies also point to osteocalcin as a new regulator of pancreatic insulin production and glucose metabolism. Bone remodeling is now believed to affect energy metabolism through uncarboxylated osteocalcin secreted by osteoblasts (Ferron et al., 2010; Fulzele et al., 2010; Rosen and Motyl, 2010; Confavreux, 2011). Systemic conditions such as diabetes and obesity directly influence bone metabolism. Adipose tissue and the adipokines (i.e., leptin) secreted by adipocytes affect bone mass. Expression of the ESP gene, which encodes a tyrosine phosphatase dephosphorylating the insulin receptor, is exclusive to osteoblasts and regulates glucose homeostasis and adiposity through controlling the osteoblastic secretion of osteocalcin (Zee et al., 2012). An undercarboxylated form of osteocalcin acts on the pancreas increasing insulin secretion, sensitivity, and β-cell proliferation (Schwetz et al., 2012). Interestingly, osteocalcin deficiency in knockout mice leads to decreased insulin and adiponectin secretion, insulin resistance, higher serum glucose levels, and increased adiposity (Wolf, 2008). These studies highlight the importance of bone in the regulation of systemic glucose homeostasis. Whether bone has thus far unexplored endocrine roles remains to be determined (Schwetz et al., 2012).

Despite these recent advances, we still know very little about glucose-sensing, transport, and metabolism in the cells of articular cartilage and bone compared to other tissues. We also know very little about how insulin and GF-1 exert overlapping roles in physiologic processes in skeletal tissues. Recent studies have identified previously unrecognized skeletal actions of insulin, which suggests that insulin-responsive bone cells participate in the regulation of global energy homeostasis (Fulzele and Clemens, 2012). Globally, there are increasing numbers of patients with insulin resistance, type 2 diabetes mellitus, and osteoporosis. Obesity, insulin resistance, and diabetes are global health issues and have major impact on bone and joint health. Further research is needed to expand our understanding of glucosensing and glucose signaling in osteoblasts, osteoclasts, and chondrocytes in health and disease. We are learning more about glucose metabolism in bone cells but the equivalent information in cartilage is largely lacking. This knowledge is important may reveal new therapeutic targets and pathways for treating metabolic diseases of articular cartilage and bone. Increasing our knowledge of these fundamental processes and the links between diabetes and bone diseases is likely to reveal new therapeutic targets for treating bone and joint disorders.

## Conflict of Interest Statement

The author declares that this review article was prepared in the absence of any commercial or financial relationships that could be construed as a potential conflict of interest.
